# PD-1单抗联合化疗治疗皮下脂膜炎样T细胞淋巴瘤一例报告并文献复习

**DOI:** 10.3760/cma.j.issn.0253-2727.2021.11.013

**Published:** 2021-11

**Authors:** 双 曲, 丽昇 廖, 颖 谢, 志海 郑, 碧云 陈

**Affiliations:** 福建医科大学附属省立临床医学院，福建省立医院血液科，福州 350001 Department of Hematology, Fujian Provincial Hospital, Fujian Medical University, Fuzhou 350001, China

皮下脂膜炎样T细胞淋巴瘤（subcutaneous panniculitis-like T-cell lymphoma, SPTCL）是一种罕见的皮肤T细胞淋巴瘤，为αβ细胞毒性T细胞浸润皮下脂肪组织，在所有原发皮肤T细胞淋巴瘤中所占比例不足1％[1]。临床上SPTCL可表现为无痛性皮下结节，部分晚期患者发热、肝脾及淋巴结肿大。疾病晚期尤其是合并噬血细胞综合征（HPS）的患者进展迅速，疗效差，预后不良[2]。本文报道一例累及皮肤及皮肤外肠系膜的初诊SPTCL患者，一、二线化疗方案原发耐药，疾病进展并继发HPS，应用程序性死亡蛋白-1（programmed death-1，PD-1）单抗联合化疗获得完全缓解。

## 病例资料

患者，女，41岁，因“反复发热1个月，发现腋窝肿物2周”于2020年5月入院。患者入院前1个月无明显诱因出现反复发热，体温波动于39 °C左右。入院前2周发现左腋窝无痛性肿物，直径2 cm×3 cm，于当地医院行肿物切除，病理初步考虑“脂肪源性肿瘤”，予泼尼松20 mg口服每日1次，应用2周，甲氨蝶呤10 mg口服每周1次治疗，发热无缓解，遂就诊于我院。查体：体温38.7 °C，余无异常。实验室检查：血常规：WBC 4×10^9^/L，HGB 125 g/L，PLT 150×10^9^/L；LDH 833 IU/L（正常参考值109～245 IU/L）；肝、肾功能均正常。全身PET-CT：双侧腋窝及胸小肌深面多发高代谢结节，最大直径0.8 cm×1.0 cm，SUVmax＝2.3。全身皮下脂肪及双侧心肋膈角区脂肪密度增高伴片状影、条索影，SUVmax＝3.8。腹、盆腔肠系膜及直肠周围脂肪间隙模糊伴多发片状影、条索影，最大直径6.8 cm×7.7 cm（右上腹腔肠系膜间隙），SUVmax＝3.6。脾门高代谢结节，直径约1.2 cm，SUVmax＝2.5。左腋窝肿物病理：送检脂肪组织，呈脂膜炎样改变，间质见小至中等大小淋巴样细胞，可见核分裂象及核碎裂，异型淋巴样细胞浸润脂肪细胞（图1A），考虑为SPTCL。免疫组化：CD3（+）、CD20（−）、CD30（−）、EBER（−）、CD8（+）（图1B）、Ki-67 50％（图1C）、CD4（−）、CD5（−）、CD7（−）、CD2（+）、TIA-1（+）（图1D）、CD56（−）、MUM1（+）、ALKp80（−）。分子病理提示TCR重排阳性。骨髓穿刺细胞形态学及骨髓流式细胞术、活检未见异常。

**图1:**
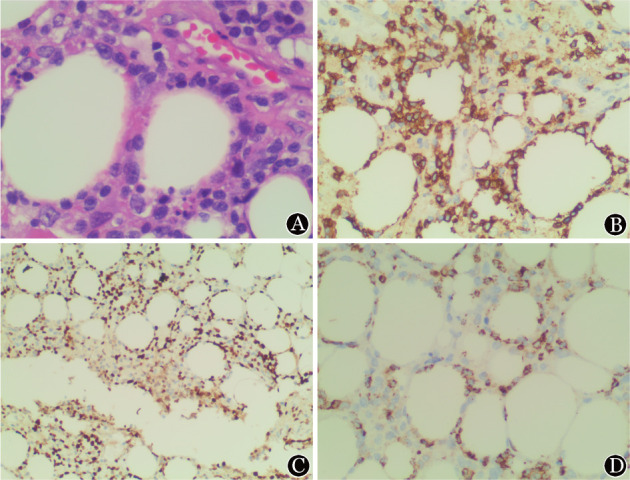
患者2020年4月左侧腋窝皮下肿物组织病理HE染色及免疫组化染色（×400） A：皮下结节HE染色示脂肪组织中淋巴细胞增生，散在、成簇及灶性分布，胞体小至中等，部分围绕脂肪细胞排列；B：CD8免疫组化染色阳性；C：免疫组化Ki-67 50％；D：TIA-1免疫组化染色阳性

患者诊断为SPTCL ⅣB期。诱导治疗采用剂量调整的ECDOP方案：环磷酰胺 1700 mg，1 g/m^2^，静脉滴注，第1天；脂质体阿霉素50 mg，静脉滴注，第1天；长春新碱 2 mg，静脉推注，第1天；地塞米松15 mg，静脉滴注，第1～5天；依托泊苷100 mg，静脉滴注，第3～5天。患者化疗1个疗程后发热缓解，但化疗间歇期左肩部新发肿物。复查血LDH 414 IU/L，彩超：左肩皮下脂肪层回声异常，结合病史考虑SPTCL可能。胸部、腹部CT：全身皮下脂肪及心肋膈角区脂肪密度增高，部分较前增多，部分较前稍减少；腹、盆腔肠系膜及直肠周围脂肪间隙部分稍增多。2020年6月11日予患者ECDOP方案（剂量同前）联合西达本胺30 mg口服每周2次。化疗间歇期新发右肩及双下肢胫前皮下结节。将组织活检病理外送至外院会诊，结论仍为SPTCL。外送组织蜡块完善淋巴瘤二代基因测序，因组织大小有限未提取成功。2020年7月6日予Gemox方案（吉西他滨1.7 g，静脉滴注，第1天；奥沙利铂170 mg，静脉滴注，第1天）联合硼替佐米2.6 mg，皮下注射，每周1次×4周，并继续西达本胺口服。患者化疗后造血恢复并出院，化疗间歇期再次发热，体温最高39.0 °C，双下肢皮下结节增大，血常规WBC 0.6×10^9^/L，PLT 10×10^9^/L。血LDH 382 IU/L，铁蛋白>1500 µg/L（正常参考值23.9～336.2 µg/L），纤维蛋白原0.9 g/L，骨髓细胞形态学：可见组织细胞噬血现象，吞噬血小板及有核红细胞、粒细胞（图2）。骨髓流式细胞术未见淋巴瘤细胞。胸部、全腹CT检查：左上肩部局部病灶密度较前稍增浓，腹腔内局部肠系膜间隙病灶较前稍多，提示病情进展。送检外周血HLA配型，未找到HLA相合异基因造血干细胞供者。2020年8月13日予PD-1单抗（信迪利单抗200 mg，第0天）+ICE方案（异环磷酰胺7.9 g，静脉滴注，第1天；卡铂0.8 g，静脉滴注，第2天；依托泊苷150 mg，静脉滴注，第1～3天）化疗，化疗后体温正常，血常规恢复，复查骨髓穿刺示噬血现象消失。2个疗程后皮下结节逐渐消失，4个疗程后复查PET-CT提示部分缓解（PR）。后因患者ICE方案化疗后骨髓抑制严重，后续治疗改为PD-1单抗（信迪利单抗）200 mg，静脉滴注，每3周1次联合西达本胺口服化疗4个疗程，复查PET-CT提示完全缓解（CR）。PD-1单抗使用期间未出现发热、过敏性皮疹、免疫性肝炎等不良反应。补做皮下结节免疫组化，结果示淋巴瘤细胞PD-L1表达阳性（图3）。

**图2:**
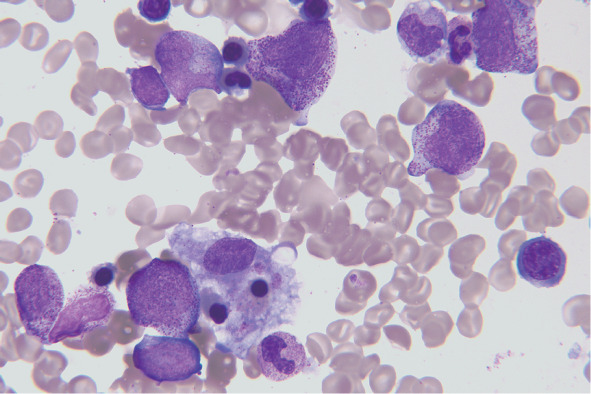
患者2020年8月骨髓细胞形态学（瑞氏染色，×100）

**图3:**
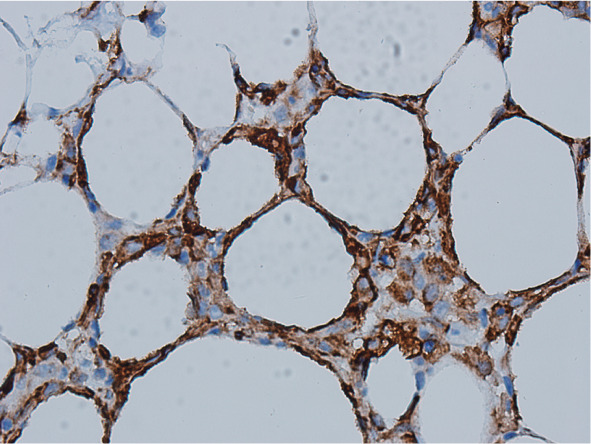
左侧腋窝皮下肿物组织病理免疫组化染色示PD-L1表达阳性（×400）

## 讨论及文献复习

1991年Gonzalez等[3]首次提出αβ和γδ表型的脂膜炎样T细胞淋巴瘤的概念，上述两种表型淋巴瘤临床表现及预后截然不同，2008年WHO将侵袭性更强的γδT细胞淋巴瘤从SPTCL中去除[1]。典型的SPTCL病理表现为皮下脂肪呈斑片状脂膜炎改变，脂肪细胞被大量小至中等T淋巴细胞围绕。T细胞表型为CD56^−^CD4^−^CD8^+^，部分可有穿孔素、TIA-1、颗粒酶等细胞毒性T细胞的标志，相对于脂膜炎有更高的Ki-67细胞增殖指数（>25％），CD2、CD5和CD7抗原丢失[4]–[5]。大约20％患者易合并自身免疫性疾病，临床表现为多发皮下结节和斑块，常分布于四肢及躯干。皮损不侵犯表皮和真皮层，因此常表现为无痛性结节，皮肤溃疡少见。本例患者以发热、无痛性皮下结节为首发症状，PET-CT结果提示疾病不仅局限于皮肤，脾门淋巴结、腹盆腔肠系膜和脂肪间隙均广泛受累，在SPTCL中更少见，目前仅检索到数例累及皮肤外肠系膜等的报道[5]–[7]。

SPTCL罕见，目前尚无标准的一线治疗方案。SPTCL早期病灶局限，治疗方案包括局部放疗、免疫抑制剂尤其是环孢素A及化疗[8]–[9]。不伴HPS的SPTCL进展缓慢，虽然目前缺乏大宗研究确定最佳的一线治疗方案，两项回顾性研究显示糖皮质激素联合甲氨蝶呤或环孢素A可作为一线推荐[4],[10]。此外，国外有病例报道显示作为组蛋白去乙酰化酶抑制剂的表观遗传学药物单药用于SPTCL亦有较好的疗效[11]–[12]。

对于晚期无法对免疫抑制剂快速起效的SPTCL患者，多药联合化疗不失为最佳选择。最常用的化疗方案为CHOP或类CHOP方案，一线CR率67％[4]。另有多篇报道显示强化方案HyperCVAD、BEACOP或BEAM序贯自体造血干细胞移植有望进一步提升患者疗效[13]。目前SPTCL的发病机制尚不清楚，20％患者伴HPS，进展迅速、易耐药，是主要死亡原因之一[4]。有研究显示60％～85％ SPTCL患者存在T细胞免疫球蛋白黏蛋白分子-3（T-cell immunoglobulin mucin 3，TIM-3）功能缺失，导致T细胞及巨噬细胞激活，引起大量细胞因子分泌[14]–[16]。Ruxolitinib作为JAK1/JAK2抑制剂曾在一例SPTCL继发HPS中有效[17]。尽管如此，目前这类患者临床治疗难度大，改善预后的方案十分有限，个别病例尝试异基因造血干细胞移植[4],[6]。

PD-1单抗作为免疫检查点抑制剂在血液肿瘤中已取得显著的疗效，尤其对于霍奇金淋巴瘤、原发纵隔大B细胞淋巴瘤[17]–[20]。对于T细胞淋巴瘤，基础研究显示PD-1单抗和表观遗传学药物在体外具有协同作用[21]–[22]。组蛋白去乙酰化酶抑制剂可上调PD-L1表达，提高PD-1单抗疗效。针对难治复发NK/T细胞淋巴瘤，中山大学肿瘤防治中心启动多中心临床研究评估信迪利单抗联合西达本胺的疗效和安全性，2020年ASH口头报告显示效果显著且安全性良好[23]。本例SPTCL患者起病时累及范围广泛，对一、二线方案原发耐药，疾病进展中继发HPS，目前何种治疗方案可改善其预后尚不明确。我们尝试PD-1单抗联合化疗取得PR，序贯PD-1单抗联合西达本胺获得CR。目前国内外尚无PD-1单抗治疗SPTCL的报道，此病例仅是个案报道，尚需要扩大样本量加以验证。晚期SPTCL患者继发HPS预后不良，PD-1单抗免疫治疗可能是一种新的选择，值得进一步探索。
